# Brainstem Correlates of Pathological Laughter and Crying Frequency in ALS

**DOI:** 10.3389/fneur.2021.704059

**Published:** 2021-07-08

**Authors:** Sicong Tu, Mengjie Huang, Jashelle Caga, Colin J. Mahoney, Matthew C. Kiernan

**Affiliations:** Brain and Mind Centre, The University of Sydney, Sydney, NSW, Australia

**Keywords:** amyotrophic lateral sclerosis, pathological laughter and crying, MRI, brainstem, motor neuron disease, pseudobulbar affect

## Abstract

Pseudobulbar affect is a disorder of emotional expression commonly observed in amyotrophic lateral sclerosis (ALS), presenting as episodes of involuntary laughter, or crying. The objective of the current study was to determine the association between frequency of pathological laughter and crying (PLC) episodes with clinical features, cognitive impairment, and brainstem pathology. Thirty-five sporadic ALS patients underwent neuropsychological assessment, with a subset also undergoing brain imaging. The Center for Neurological Study Lability Scale (CNS-LS) was used to screen for presence and severity of pseudobulbar affect (CNS-LS ≥ 13) and frequency of PLC episodes. Presence of pseudobulbar affect was significantly higher in bulbar onset ALS (*p* = 0.02). Frequency of PLC episodes was differentially associated with cognitive performance and brainstem integrity. Notably pathological laughter frequency, but not crying, showed a significant positive association with executive dysfunction on the Trail Making Test B-A (*R*^2^ = 0.14, *p* = 0.04). Similarly, only pathological laughter frequency demonstrated a significant negative correlation with gray matter volume of the brainstem (*R*^2^ = 0.46, *p* < 0.01), and mean fractional anisotropy of the superior cerebellar peduncles (left: *R*^2^ = 0.44, *p* < 0.01; right: *R*^2^ = 0.44, *p* < 0.01). Hierarchical regression indicated brainstem imaging in combination with site of symptom onset explained 73% of the variance in pathological laughter frequency in ALS. The current findings suggest emotional lability is underpinned by degeneration across distinct neural circuits, with brainstem integrity critical in the emergence of pathological laughter.

## Introduction

Pseudobulbar affect (PBA) is characterized by recurrent episodes of involuntary changes in emotional expression incongruent with the social context and magnitude of evoking environmental stimulus. The episodes occur suddenly and with varying degrees of severity typically presenting as pathological laughter and/or crying (PLC). Presentation of PLC symptoms is well-documented across motor and, to a lesser extent, cognitive predominant neurodegenerative conditions (1, 2). The underlying pathophysiology of PLC is not focal, as evidenced by its clinical presence across diverse neurological conditions with disruption of the cortico-pontine-cerebellar neural network (2). Neuroimaging studies have consistently implicated abnormalities in the integrity of underlying frontal-brainstem neural circuitry believed to regulate motor control of affective emotional expression (1, 3). Whilst PLC is typically considered a secondary feature accompanying primary neurological diseases, clinical management is critical due to the disruptive and socially disabling consequences of symptoms associated with reduced quality of life and increased use of psychotropic medication (2). This is of particular relevance in amyotrophic lateral sclerosis (ALS), where the reported prevalence of PLC symptoms is reported to be present in 25–50% of patients (4). Patients with bulbar onset symptomatology appear disproportionately affected (5), possibly reflecting greater brainstem pathology within the ALS clinical spectrum (6, 7). This is consistent with an increased incidence of PLC in neurodegenerative conditions with significant atrophy impacting brainstem and cerebellum integrity (8).

The neural circuitry and pathways mediating PLC remain poorly defined but center on a disinhibition model, viewing disruption of inhibitory input in descending motor and emotion processing corticopontine pathways as the driver of symptoms (1, 9). Increasingly, however, the importance of the cognitive cerebellum (10), as a modulator of behavioral response based on sensory cues, has been proposed as a core component (1). This is consistent with previously published neuroimaging findings in ALS indicating greater abnormality in pontocerebellar white matter for patients with PLC (11, 12). Reported findings, however, are based on whole-brain analysis approaches and the association between integrity of specific brainstem structures and PLC in ALS remains unclear.

The diagnosis of pseudobulbar affect across neurological disorders is typically performed informally as part of routine clinical examination. While there are a number of diagnostic criteria proposed (13–15), none have been widely adopted (4), possibly leading to underdiagnosis. Objective clinical screening scales of affective emotional lability are available (16–18). Notably, the Center for Neurologic Study-Lability Scale (CNS-LS), a brief 7-item self-administered questionnaire has been validated in ALS patients and shown to be an accurate indicator of PLC episode frequency (16). The questionnaire has been adopted as an outcome measure in ALS clinical trials (19, 20). A cutoff of 13 has been shown to accurately predict neurologist's clinical diagnosis of pseudobulbar affect in ALS patients (16). However, such screening tools may be impacted by a range of other cognitive or behavioral symptoms affecting sensitivity.

The current study aimed to define the association between the integrity of reliably segmented brainstem structures and PLC in a well-defined cohort of sporadic ALS patients. The association with PLC as a whole as well as its individual laughter and crying components were examined, an aspect which remains absent from the existing ALS neuroimaging literature. Additionally, we examined the interaction of cognitive dysfunction and PLC. In accordance with brainstem dysfunction, which has been proposed as a key region of pathological change in the earliest stage of ALS (21), we hypothesized that reduced integrity of brainstem structures and adjoining cortico-ponto-cerebellar pathways may significantly contribute to increased severity of PLC symptoms in patients at initial clinical examination. We further hypothesized that the neural correlates of pathological laughter and crying in ALS contain unique differences, given reported distinctions in their association with clinical features (22).

## Materials and Methods

### Participants

Clinical data was retrospectively analyzed from all patients (*n* = 35) presenting with sporadic, classical forms of ALS seen at the Sydney Forefront MND Clinic between 2013 and 2015, who had completed the Center for Neurologic Study-Liability Scale (CNS-LS). The CNS-LS is a 7 item questionnaire assessing frequency of pathological laughter and crying symptoms and was administered as part of their clinical examination. Patients were categorized into PBA– and PBA+ groups according to a cutoff CNS-LS total score ≥13, respectively (16, 19). The clinical diagnosis of probable or definite ALS, and determination of regional involvement was made by an experienced neurologist (MCK), in accordance with revised El Escorial criteria (23). Patients did not present with comorbid mild and/or major neurocognitive disorders. Disease duration was calculated from date of first reported weakness. Severity of functional disability was assessed using the revised ALS functional rating scale (ALSFRS-R), a four domain disability questionnaire with scores ranging from 0 to 48, with a lower score reflecting greater disability. All participants underwent a standardized comprehensive neuropsychological assessment (for description see, Supplementary Table 1). General cognitive function was assessed using the revised Addenbrooke's Cognitive Examination (ACE-R), covering domains of attention, memory, fluency, language, and visuospatial processing, whereby a higher score indicates better performance. Raw ACE-R scores were converted into percentages to account for items not administered due to motor impairment (24). Verbal fluency was assessed for item (Letter P Items, Excluded Letter E) and categorical (Animals) naming performance (25). Visual processing speed was assessed using the Trail Making Test, the time difference between B-A components was used as a measure of executive function.

Ethical approval for this study was obtained from the University of Sydney, and the South Eastern Area Health Service ethics committees.

### MRI Acquisition

MRI data was available for 14 participants. All participants were scanned at the Brain and Mind Center using a 3T GE Discovery MR750 scanner with 12-channel head coil on the same day as clinical assessment. Whole-brain T1-weighted MRI scans were acquired using a magnetization-prepared rapid gradient echo sequence (TE/TR = 2.6/5.8 ms; flip angle = 8°; matrix size = 256 × 256, 200 slices, 1 mm^3^ isotropic). Whole-brain diffusion-weighted images were acquired using an echo-planar sequence (32 isotropic directions; b-value = 1,000 s/mm^2^; TE/TR = 68/8,400 ms; 55 slices; 2.5 mm^3^). Four additional b0 images without diffusion weighting were acquired.

### Volumetric Imaging Analysis

Each individual's structural MRI scan was processed using the FreeSurfer V.6.0 software package (https://surfer.nmr.mgh.harvard.edu), with brainstem segmentation (26). FreeSurfer brainstem segmentation was chosen for its relatively high reproducibility compared to other methods (27). T1-weighted images were first processed through the standard FreeSurfer recon-all pipeline, correcting for motion and intensity inhomogeneity, removal of non-brain tissue, and segmentation of white and gray matter. A robust Bayesian inference algorithm was then applied to detect local variations in MRI contrast within the brainstem subfield to segment and calculate volumes associated with the midbrain, pons, and medulla oblongata (26). All segmentations for the brainstem were visually checked for accuracy. Total brain volume was calculated through FreeSurfer, and brainstem volumes were normalized accordingly, correcting for interindividual differences.

### Diffusion Imaging Analysis

Participant's diffusion-weighted scans were processed using tools from the FMRIB Software Library (https://fsl.fmrib.ox.ac.uk/fsl). All scans were corrected for head motion and eddy currents followed by the removal of all non-brain voxels (28, 29). DTIFIT was used to apply a diffusion tensor model at each voxel, generating three eigenvalues (L1, 2, 3) for calculating whole-brain fractional anisotropy (FA) maps. Region of interest masks for the cerebellar peduncles (superior, middle, inferior) were generated from the John Hopkins University ICBM DTI-81 stereotaxic white matter atlas. FSL-FLIRT (30) was used to independently register each individual's mean b0 to T1 image, and T1 image to MNI standard space. Resulting matrices were combined and inverted to apply a two-stage affine registration, transforming cerebellar peduncle labels to native diffusion space. Labels were visually inspected for accuracy and mean FA values were extracted for each region.

### Statistical Analyses

Statistical analyses examining cognitive performance and brainstem MRI correlates of emotional lability frequency in ALS were carried out using SPSS V.21.0. The Shapiro-Wilk test was used to test normality. Two-tailed independent samples *t*-test was used to compare clinical features and cognitive performance between PBA– and PBA+ ALS cohorts. Two-tailed Pearson's correlation was used to examine correlations between CNS-LS with executive dysfunction and imaging metrics. Results were adjusted for multiple comparisons using Bonferroni correction. Hierarchical multiple regression was conducted to examine whether brainstem neuroimaging significantly contributed to explaining emotional lability variance in ALS patients beyond clinical features. In all analyses, *p* < 0.05 were considered to be significant.

## Results

### Patient Data and Demographics

ALS participants were categorized into PBA– (*n* = 16) and PBA+ (*n* = 19) patient cohorts based on frequency of emotional lability episodes as reflected by CNS-LS total score (≥13), respectively (Table 1). Patient cohorts were well-matched across all demographic and clinical characteristics (*p* > 0.2) with the exception of site of initial symptom onset. The PBA+ cohort demonstrated significantly greater incidence of bulbar involvement (*p* = 0.02). There were no significant group differences across neuropsychological measures of general cognition (ACE-R, *p* > 0.3), verbal fluency (Letter P Items, *p* = 0.96; Animals, *p* = 0.8; Excluded Letter E, *p* = 0.17), and visual attention (Trail Making Test, *p* > 0.6). Mean total score on the ACE-R in PBA– and PBA+ cohorts was within 2 standard deviation of normative age scores (mean = 94.45; SD = 3.2), and above the upper limit cut-off of 88 for suspected dementia. Mean performance on the Trail Making Test A was within 1 standard deviation of normative age performance in the PBA– (mean = 31.32; SD = 6.96) and PBA+ (mean = 33.84; SD = 6.69) cohorts. Mean performance on the Trail Making Test B was within 2 standard deviation of normative age performance for the PBA– cohort (mean = 64.58; SD = 18.59) and 3 standard deviation for the PBA+ cohort (mean = 67.12; SD = 9.31).

**Table 1:** Demographic characteristics and clinical profile of ALS patients with and without pseudobulbar affect as defined by CNS-LS score (≥13).

	**ALS PBA– (*n* = 16)**	**ALS PBA+ (*n* = 19)**	**PBA– vs. PBA+ *P* value**
Gender (M/F)	13M, 3F	12M, 7F	0.29
Handedness (L/R/B)	16R	2L, 16R, 1B	0.25
Age (y.o)	63.3 (14.8)	65.6 (11)	0.6
Education (yrs)	12.3 (2.4)	12.1 (3.1)	0.84
Disease Duration (months)	45.3 (35.6)	34.4 (27.4)	0.32
ALSFRS-R	41.5 (5.1)	38.7 (7.1)	0.21
Site of Initial Symptom Onset	Bulbar 15 Limb	8 Bulbar 11 Limb	0.02^*^
CNS-LS			
Total (/35)	8.7 (1.7)	18 (3.3)	<0.01^*^
Laughter (/20)	4.9 (1.2)	10.4 (3.6)	<0.01^*^
Crying (/15)	3.8 (1.2)	7.6 (2.3)	<0.01^*^
ACE-R			
Total (%)	90.4 (11.4)	89.2 (9)	0.48
Attention (%)	93.6 (7.7)	96.2 (5.1)	0.49
Memory (%)	87.1 (16.7)	86.2 (14.9)	0.74
Fluency (%)	79.2 (18.1)	75.9 (19.9)	0.58
Language (%)	93.8 (10.2)	90.8 (9.8)	0.34
Visuospatial (%)	97.1 (5.7)	94.2 (8.2)	0.34
Verbal Fluency			
Letter P Items	3.5 (2.4)	3.5 (2.7)	0.96
Animals	2.7 (1.7)	2.9 (2.2)	0.8
Excluded Letter E	4 (1.5)	5.9 (4.3)	0.17
Trail Making Test			
A	33.9 (18.7)	32.3 (15.5)	0.79
B	85.7 (60.8)	93.1 (67.9)	0.76
B-A	51.8 (54.4)	60.8 (55.2)	0.66

*Mean and standard deviation*.

* *Indicates significant at p < 0.05; Center for Neurologic Study-Liability Scale (CNS-LS)*.

### Clinical Correlates of Emotional Lability

Emotional lability frequency on the CNS-LS was significantly correlated with executive dysfunction on the Trail Making Test. A significant positive correlation was observed between impaired executive function, reflected by impaired task-switching inhibition on the Trail Making test (B-A), with CNS-LS total score (*R*^2^ = 0.13, *p* = 0.04) and laughter score (*R*^2^ = 0.14, *p* = 0.04). CNS-LS crying score showed no significant correlation with executive dysfunction (*p* = 0.29). No significant correlations were observed with measures of Verbal Fluency (*p* > 0.07).

### Brainstem Correlates of Emotional Lability

**Figure 1:**
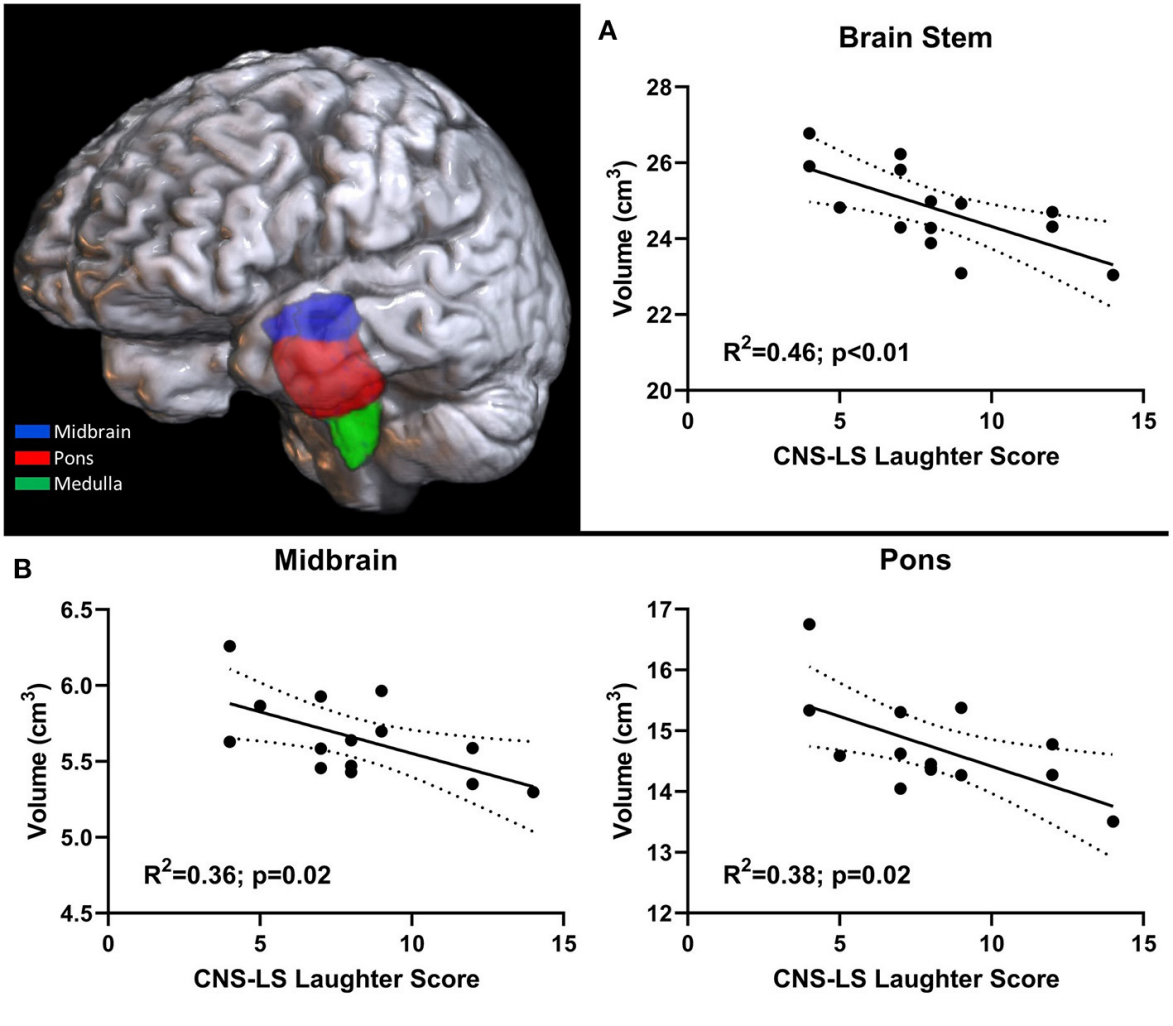
3D rendering of brainstem segmentation in an individual participant. Significant correlation between pathological laughter severity and **(A)** total brainstem volume, **(B)** midbrain and pons volume in ALS patients.

Structural scans of ALS participants with an MRI scan (*n* = 14; Supplementary Table 2) were processed in Freesurfer to reliably segment the brainstem into midbrain, pons, and medulla (Figure 1). Resulting brainstem segmentations were corrected for total-intracranial volume and correlated with CNS-LS scores. CNS-LS total score and laughter score demonstrated significant negative correlations with brainstem volume, indicating increased frequency of emotional lability episodes with reduced brainstem volume. CNS-LS total score significantly correlated with volume of the whole brainstem (*R*^2^ = 0.38, *p* = 0.02). CNS-LS laughter score demonstrated a similar, but stronger, correlation with volume of the whole brainstem (*R*^2^ = 0.46, *p* < 0.01), midbrain (*R*^2^ = 0.36, *p* = 0.02), and pons (*R*^2^ = 0.38, *p* = 0.02), but not medulla (*R*^2^ <0.01, *p* = 0.77). No significant correlations were observed for CNS-LS crying score with volume of the whole brainstem (*R*^2^ = 0.19, *p* = 0.11), midbrain (*R*^2^ = 0.2, *p* = 0.32), pons (*R*^2^ = 0.16, *p* = 0.16), and medulla (*R*^2^ = 0.08, *p* = 0.32).

**Figure 2:**
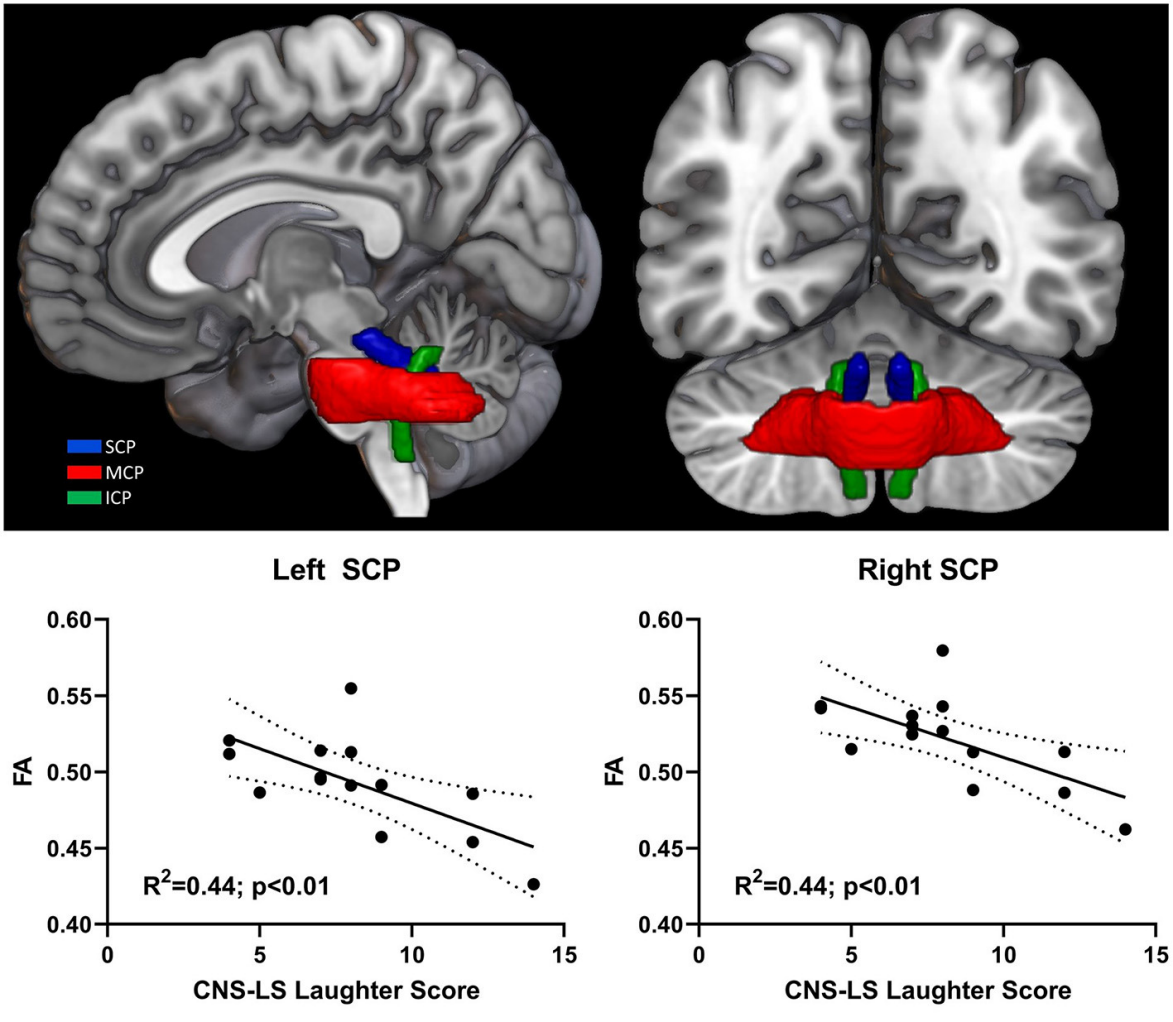
3D rendering of cerebellar peduncle masks on the MNI152 standard brain. Significant correlation between pathological laughter severity and mean fractional anisotropy of middle cerebellar peduncle in ALS patients. Cerebellar peduncles: superior (SCP); middle (MCP); inferior (ICP).

Mean FA values were extracted for cerebellar peduncles (superior, middle, inferior) across both hemispheres for all participant scans and examined for correlation with CNS-LS scores (Figure 2). CNS-LS total score and laughter score demonstrated a similar negative correlation with white matter integrity in the left and right superior cerebellar peduncles, indicating increased frequency of emotional lability episodes with reduced FA. CNS-LS total score significantly correlated with mean FA of the superior cerebellar peduncles (left: *R*^2^ = 0.36, *p* = 0.02; right: *R*^2^ = 0.34, *p* = 0.02). CNS-LS laughter score demonstrated a similar, but stronger, correlation with mean FA of the superior cerebellar peduncles (left: *R*^2^ = 0.44, *p* < 0.01; right: *R*^2^ = 0.44, *p* < 0.01). No significant correlations were observed for CNS-LS crying score (Supplementary Table 3). The same pattern of results was observed for radial diffusivity (Supplementary Table 3).

### Hierarchical Regression of Emotional Lability with Brainstem Integrity

Separate hierarchical regression was performed for CNS-LS total and laughter score examining the relationship with brainstem MRI predictor variables, while controlling for site of initial symptom onset (Table 2). Tests of collinearity indicated multicollinearity among predictor variables in both regression models was not of concern (Tolerance > 0.86; VIF < 1.16). For CNS-LS total score, the regression model indicated that the combination of midbrain and pons volume, mean FA of left/right superior cerebellar peduncles, and site of initial symptom onset, accounted for 67% of variance. The addition of volumetric and diffusivity metrics of correlated brainstem regions of interest significantly accounted for additional variance of CNS-LS total (Volume, 21%; FA, 17%; *p* < 0.05). A similar predictive pattern was observed for CNS-LS laughter score. The regression model for CNS-LS laughter score, using the same predictor variables, accounted for 73% of variance. The addition of volumetric and diffusivity metrics accounted for additional variance in laughter score (Volume, 29%; FA, 22%; *p* < 0.03).

**Table 2:** Hierarchical regression analysis for brainstem MRI variables predicting CNS-LS total and laughter scores.

**Variable**	**β**	***t***	**sr^**2**^**	**R**	***R*^**2**^**	**ΔR^**2**^**
**CNS-LS Total**						
Step 1				0.54	0.29	0.29
Site of Initial Symptom Onset	−0.54	−2.22^*^	0.29			
Step 2				0.71	0.5	0.21
Site of Initial Symptom Onset	−0.41	−1.85	0.16			
Midbrain + Pons Volume	−0.48	−2.14^*^	0.21			
Step 3				0.82	0.67	0.17
Site of Initial Symptom Onset	−0.37	−1.97	0.12			
Midbrain + Pons Volume	−0.36	−1.84	0.11			
Superior Cerebellar Peduncle FA	−0.43	−2.26^*^	0.17			
**CNS-LS Laughter**						
Step 1				0.47	0.22	0.22
Site of Initial Symptom Onset	−0.47	−1.83	0.22			
Step 2				0.71	0.51	0.29
Site of Initial Symptom Onset	−0.32	−1.44	0.09			
Midbrain + Pons Volume	−0.56	−2.52^*^	0.29			
Step 3				0.86	0.73	0.22
Site of Initial Symptom Onset	−0.28	−1.62	0.07			
Midbrain + Pons Volume	−0.42	−2.4^*^	0.15			
Superior Cerebellar Peduncle FA	−0.5	−2.93^**^	0.23			

*n = 14;*

* *p < 0.05;*

** *p < 0.01*.

## Discussion

In the current study, 54% of ALS patients met the clinical cut-off for pseudobulbar affect on the CNS-LS (16, 19). This prevalence rate in seemingly unselected ALS patients is high relative to previously published population-based CNS-LS incidences of 28–45% (2, 22, 31), though may relate to our center's expertise in cognitive dysfunction impacting patient referral. Consistent with previous findings, higher incidence of an initial bulbar site of symptom onset was observed in PBA+, relative to PBA–, ALS patients (22). Analyses of cognitive and brainstem associations with the CNS-LS indicated significant correlations only with pathological laughter frequency. Increased frequency of pathological laughter correlated with increased executive dysfunction on the Trail Making Test (B-A), reduced volume of the midbrain and pons, and reduced white matter integrity in bilateral superior cerebellar peduncles. Hierarchical regression demonstrated that quantitative brainstem imaging metrics provided a useful addition for explaining the clinical variance in severity of pathological laughter frequency in ALS. The current findings expand the sparse PLC neuroimaging literature in ALS (11, 12) and suggests brainstem integrity as a potentially useful independent marker of PLC frequency. Furthermore, this may offer a useful outcome measure for therapeutic trials across a range of neurological disorders associated with PLC.

Emotional expression in humans is a complex system that has evolved from automated motor and somatosensory responses from evoking environmental stimuli to involve complex cognitive deliberations including our experiences and beliefs (32). Expression is therefore finely modulated by a distributed network of ascending and descending cortical and subcortical networks involving phylogenetically older structures, such as the brainstem and cerebellum, integrative subcortical relay structures, such as the thalamus and hypothalamus, and higher order cortical motor and frontal processing centers (33). The brainstem and cerebellum are proposed to play a primary role in the modulation of emotion and emotional behavior, in particular the subjective experience of both positive and negative emotions (33, 34). In essence, PLC is characterized as an emotional modulatory dysfunction resulting in a sudden change in emotional response inconsistent with internal state. Disruption to the integrity of descending corticopontine and ascending cortico-cerebellar pathways, as reported in ALS (12, 35, 36), may therefore represent a more specific factor underlying PLC severity relative to broader dysfunction in higher order cortical processing. The current findings in sporadic ALS patients further support this notion and is consistent with the reported neural signature of PLC across neurological conditions consistently implicating a significant association with brainstem lesions, pons volume, and cerebellar white matter abnormality (4). Variability in the localization of PLC associated cerebellar peduncle (superior, middle, inferior) abnormality in ALS (11, 12) suggests it is the integrity of the input/output circuity as a whole that is important to ensure appropriate scaling or inhibition of emotional expression relative to social context (1, 37).

A novel component of the current study was the independent investigation of laughter and crying frequency components of the CNS-LS in combination with multi-modal neuroimaging in ALS, which appears overlooked in the existing literature (11, 38). The current findings indicated stronger associations between CNS-LS laughter frequency with brainstem imaging metrics, compared to total score. Notably, crying frequency did not show any significant associations with either executive dysfunction or brainstem imaging. This finding may be driven by a gender bias within the patient cohort of predominantly male patients. A previous population-based study of CNS-LS in ALS (22) indicated a gender bias in patients classified with pseudobulbar affect, whereby 64.7% of women cried predominantly, whereas 64.4% of men laughed predominantly. One possible explanation, however, is the developmental shift of crying in adults as a mechanism for emotional rather than physical solace, involving greater distributed cortical inhibitory processes. In contrast, laughter remains a more primal and spontaneous response critical for social inclusion (39) and a reliable cross-cultural indicator of relationship quality (40). Consequently, the pathological laughter observed in ALS patients may be primarily driven by dysfunction across phylogenetically older motor and autonomic brainstem and cerebellar structures. Nevertheless, laughter and crying are highly interlinked and in the case of PLC can begin as laughter and transition into crying within the same clinical episode. This is supported by working models of an underlying cortico-pontine-cerebellar neural circuitry of PLC which does not appear to draw a distinction between pathological laughter and crying (1, 9).

### Limitations of Study

The current findings used historical single-center clinical data. ALS is a clinically heterogenous condition and the generalizability of the current findings requires further multi-center validation. Patient sample size constrained the scope of neuroimaging analyses and previously published recommendations for gender and depression stratification were absent (22). Adjusting for depressive symptoms is unlikely to have influenced the current neuroimaging findings as no patients had a clinical history of major depressive disorder. Furthermore, the previous validation study of the CNS-LS in ALS indicated depressive symptoms only accounted for 6% of variance in CNS-LS scores (16). While the CNS-LS provides an objective measure of PLC severity, this is limited to frequency. Tools for interrogating the qualitative nature of PLC is needed to better disentangle behavioral, physiological, and subjective contributions driving clinical symptoms in ALS (41).

### Conclusions and Future Directions

In conclusion, ALS presents an ideal model to advance current understanding of the underlying neural mechanisms of PLC. There is a significant association between reduced integrity of brainstem volume and diffusivity of cerebellar peduncles with emotional lability, in particular pathological laughter frequency. Prospective studies using population-based ALS cohorts should consider including more fine-grained cognitive and behavioral phenotyping to disentangle their contributions to the clinical presentation of PLC (42). Further investigation of laughter and crying components with a larger patient sample is necessary to demonstrate whether there is a distinction in neural circuitry of PLC in ALS. Patients with frontotemporal lobar degeneration, which holds pathological overlap with ALS, have also been associated with high levels of pathological laughter (43), suggesting a shared pathological substrate that would be of interest in future comparative clinical imaging studies. Pharmaceutical agents modulating serotonin/dopamine deficiency, glutamate excess, and sigma type 1 receptor dysfunction have shown varying degrees of efficacy in reducing frequency of PLC episodes (1, 44). Future investigation of brainstem-derived neurochemical profiles may help drive therapeutic advances of more efficacious treatment options for managing PLC in ALS patients.

## Data Availability Statement

The raw data supporting the conclusions of this article will be made available by the authors, without undue reservation.

## Ethics Statement

The studies involving human participants were reviewed and approved by Human Research Ethics Committee, the University of Sydney & South East Sydney Local Health District Ethics Committee. The patients/participants provided their written informed consent to participate in this study.

## Author Contributions

ST, JC, and CM contributed to study conception and design. ST, MH, JC, and MK contributed to data collection and organization. ST and MH performed the statistical analyses. ST wrote the first draft of the manuscript. All authors contributed to manuscript revision, read, and approved final manuscript submission.

## Conflict of Interest

The authors declare that the research was conducted in the absence of any commercial or financial relationships that could be construed as a potential conflict of interest.
