# Supplementary material for: Brainstem Correlates of Pathological Laughter and Crying Frequency in ALS
Source: Front Neurol. 2021 Jul 8;12:704059. doi: 10.3389/fneur.2021.704059 (PMC8296641; doi:10.3389/fneur.2021.704059)
Supplement: Supplementary file 1 [file Table_1.DOCX]

**Supplementary Table 1.** Description of neuropsychological tasks administered.

| **Neuropsychological Test** | **Task Components** |
| --- | --- |
| Addenbrooke Cognitive Exam –Revised (ACE-R)  (Mioshi et al., 2006)^1^ | The ACE-R is a general screening measure of cognition scored out of 100 and contains sub-components assessing: attention and orientation, memory, verbal fluency, language, and visuospatial skills. The memory subtest score comprises (1) recall after brief distraction of a three-item list, (2) recall of a seven-item name and address on the third learning trial, (3) delayed recall and recognition of the name and address, (4) recall of the names of 4 specified current and previous politicians. |
| Verbal Fluency  (Abrahams et al., 2000)^2^ | Participants start with a generation condition whereby they are asked to write down as many words for a given condition within 60 seconds.  Letter P Items: words beginning with the letter P  Excluded Letter E: words that do not contain the letter E  Category (Animals): items belonging to the ‘animal’ category  Following a delay, participants complete a control condition where they copied the previously generated words to control for existing motor impairment. Performance was scored as a fluency index (fi) representing the average time taken to think of each item. fi=(time for generation condition)-(time for control condition) |
| Trail-making Test  (Tombaugh, 2004)^3^ | The Trail Making Test is a neuropsychological test of visual attention and task switching. It consists of two parts (A, B) in which the subject is instructed to connect a set of 25 dots as quickly as possible while maintaining accuracy. The test can provide information about visual search speed, scanning, speed of processing, mental flexibility, as well as executive functioning. |

**References:**

1. Mioshi E, Dawson K, Mitchell J, Arnold R, Hodges JR. The Addenbrooke's Cognitive Examination Revised (ACE-R): a brief cognitive test battery for dementia screening. Int J Geriatr Psychiatry 2006;21:1078-1085.

2. Abrahams S, Leigh PN, Harvey A, Vythelingum GN, Grise D, Goldstein LH. Verbal fluency and executive dysfunction in amyotrophic lateral sclerosis (ALS). Neuropsychologia 2000;38:734-747.

3. Tombaugh TN. Trail Making Test A and B: normative data stratified by age and education. Arch Clin Neuropsychol 2004;19:203-214.

**Supplementary Table 2.** Demographic characteristics and clinical profile of ALS patients with an MRI scan. Mean and standard deviation.

|  | **ALS**  **(n=14)** |
| --- | --- |
| **Gender (M/F)** | 12M, 2F |
| **Handedness (L/R/B)** | 12R |
| **Age (y.o)** | 61.8 (10.5) |
| **Education (yrs)** | 11.7 (2.6) |
| **Disease Duration (months)** | 32.2 (27.4) |
| **ALSFRS-R** | 43.7 (3.1) |
| **Site of Initial Symptom Onset** | 4 Bulbar  10 Limb |
| **CNS-LS** |  |
| **Total (/35)** | 14.4 (5.1) |
| **Laughter (/20)** | 6.2 (2.5) |
| **Crying (/15)** | 8.1 (2.9) |
| **ACE-R** |  |
| **Total (%)** | 93.3 (5.6) |
| **Attention (%)** | 96.1 (5.6) |
| **Memory (%)** | 90.3 (9.7) |
| **Fluency (%)** | 84.5 (14.4) |
| **Language (%)** | 94.8 (7.9) |
| **Visuospatial (%)** | 97.5 (4.8) |
| **Verbal Fluency** |  |
| **Letter P Items** | 2.9 (2.1) |
| **Animals** | 2.3 (1.2) |
| **Excluded Letter E** | 5.4 (3) |
| **Trail-making Test** |  |
| **A** | 31.3 (10.4) |
| **B** | 80.5 (49.8) |
| **B-A** | 49.1 (40.4) |

* Center for Neurologic Study – Liability Scale (CNS-LS)

**Supplementary Table 3.** Correlation between mean diffusivity measures of cerebellar peduncle integrity and CNS-LS scores.

| **Fractional Anisotropy (FA)** | | | | |
| --- | --- | --- | --- | --- |
| **CNS-LS Total Score** | **Cerebellar Peduncle** | **Hemisphere** | **R^2^** | **P-value** |
|  | Superior | L | **0.36** | **0.01*** |
|  | Superior | R | **0.34** | **0.01*** |
|  | Middle | L/R | 0.09 | 0.29 |
|  | Inferior | L | 0.19 | 0.12 |
|  | Inferior | R | 0.17 | 0.14 |
| **CNS-LS Laughter Score** |  |  |  |  |
|  | Superior | L | **0.44** | **<0.01*** |
|  | Superior | R | **0.44** | **<0.01*** |
|  | Middle | L/R | 0.19 | 0.12 |
|  | Inferior | L | 0.26 | 0.07 |
|  | Inferior | R | 0.23 | 0.08 |
| **CNS-LS Crying Score** |  |  |  |  |
|  | Superior | L | 0.18 | 0.13 |
|  | Superior | R | 0.16 | 0.16 |
|  | Middle | L/R | 0.01 | 0.72 |
|  | Inferior | L | 0.08 | 0.33 |
|  | Inferior | R | 0.07 | 0.36 |
| **Radial Diffusivity (RD)** | | | | |
| **CNS-LS Total Score** | **Cerebellar Peduncle** | **Hemisphere** | **R^2^** | **P-value** |
|  | Superior | L | **0.52** | **<0.01*** |
|  | Superior | R | **0.59** | **<0.01*** |
|  | Middle | L/R | 0.3 | 0.04 |
|  | Inferior | L | 0.02 | 0.66 |
|  | Inferior | R | 0.16 | 0.16 |
| **CNS-LS Laughter Score** |  |  |  |  |
|  | Superior | L | **0.48** | **<0.01*** |
|  | Superior | R | **0.54** | **<0.01*** |
|  | Middle | L/R | 0.24 | 0.08 |
|  | Inferior | L | 0.03 | 0.58 |
|  | Inferior | R | 0.21 | 0.1 |
| **CNS-LS Crying Score** |  |  |  |  |
|  | Superior | L | 0.25 | 0.07 |
|  | Superior | R | 0.23 | 0.08 |
|  | Middle | L/R | 0.28 | 0.05 |
|  | Inferior | L | <0.01 | 0.82 |
|  | Inferior | R | 0.07 | 0.35 |

*Significant at Bonferroni-corrected threshold, p=0.01
